# Quantitative investigation of linear arbitrary polarization in an APPLE-II undulator

**DOI:** 10.1107/S1600577518001960

**Published:** 2018-02-22

**Authors:** Matthew Hand, Hongchang Wang, Francesco Maccherozzi, Marco Apollonio, Jingtao Zhu, Sarnjeet S. Dhesi, Kawal Sawhney

**Affiliations:** a Diamond Light Source, Harwell Science and Innovation Campus, Didcot OX11 0DE, UK; b Institute of Precision Optical Engineering, Tongji University, Shanghai 200092, People’s Republic of China

**Keywords:** polarization, multilayers, synchrotron radiation, undulators

## Abstract

Insertion devices are utilized at synchrotron radiation facilities around the world for their capability to provide a high-brilliance X-ray beam. APPLE-II type undulators are especially important for their capacity to switch between a variety of photon beam polarization states. A high-precision soft X-ray polarimeter has been used to investigate the polarization calibration of an APPLE-II undulator (period length λ_u_ = 64 mm) installed on beamline I06 at Diamond Light Source. Systematic measurement of the beam polarization state at a range of linear arbitrary angles has been compared with the expected result for a given set of undulator gap and row phase parameters calculated from theory. Determination of the corresponding Stokes–Poincaré parameters from the measured data reveals a discrepancy between the two. The limited number of energy/polarization combinations included in the undulator calibration tables necessitates the use of interpolated values for the missing points which is expected to contribute to the discrepancy. However, by modifying the orbit of the electron beam through the undulator by at least 160 µm it has been found that for certain linear polarizations the discrepancies can be corrected. Overall, it is suggested that complete correction of the Stokes–Poincaré parameters for all linear angles would require alteration of both these aspects.

## Abstract 2

Polarization calibration of the photon beam produced by an APPLE-II undulator using a multilayer-based soft X-ray polarimeter is presented.

## 1. Introduction   

**Figure 1:**
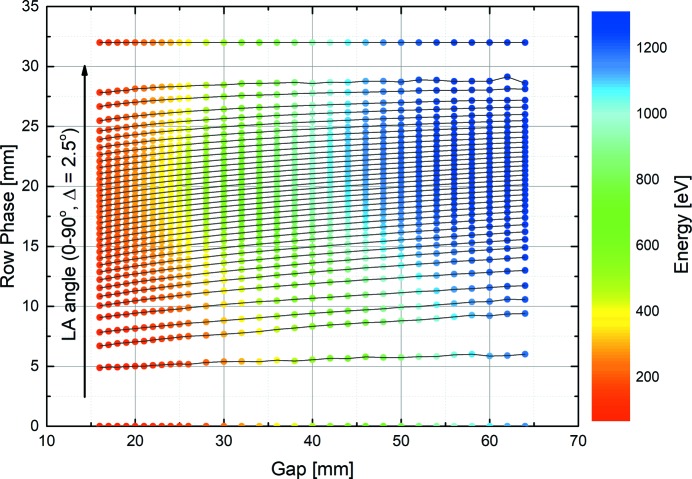
Calculated undulator parameter set covering the complete range of linear arbitrary angles. These are linearly interpolated to produce look-up tables evenly spaced in energy and LA angle for compatibility with the underlying EPICS control system used to operate the undulator.

**Figure 2:**
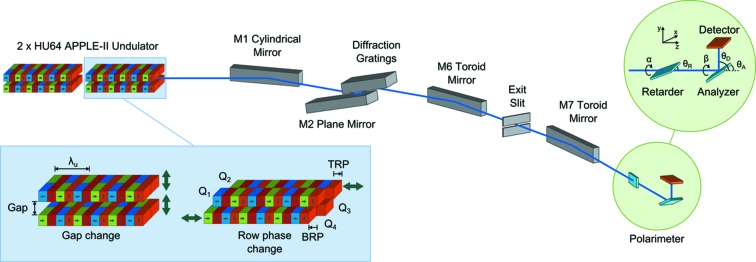
Schematic layout of the experimental setup with the soft X-ray polarimeter installed on the beamline I06 branch line. With four magnet rows (quadrants Q1–Q4), the APPLE-II undulator offers the capability to alter the row phases of quadrants Q2 and Q4 independently, referred to as top and bottom row phase (TRP/BRP) here, along with the gap between the two magnet row pairs. This provides the capability of selecting almost any polarization state across the entire energy range of the source.

Undulator sources are commonly used on beamlines at modern synchrotron radiation facilities owing to their capacity to deliver brilliant soft X-ray beams with variable polarization (Sasaki, 1994 ▸; Hwang & Yeh, 1999 ▸; Weiss *et al.*, 2001 ▸). Among the variety of undulator types available, APPLE-II helical undulators (HUs), consisting of four magnet array quadrants Q1–Q4, are widely used for their flexibility in varying the emitted radiation polarization between left-handed and right-handed circular polarization and linear polarization with arbitrary angle (which will be the focus here) by adjusting the relative position of the magnet arrays. In the latter case, the undulator parameters in question are the gap between the top and bottom array pairs, and the longitudinal offset (hereafter referred to as ‘row phase’) which for linear light is equal and opposite for diagonally opposing arrays, *i.e.* quadrants Q2 and Q4 as indicated in Fig. 2 (Longhi *et al.*, 2013 ▸). Therefore, to set linear polarization with arbitrary angle we have a two-valued two-dimensional function *F* representing the beam state which must be invertible (for energy/angle readback) (Young *et al.*, 2002 ▸),
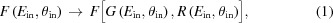


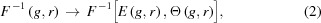
where *G* and *R* are the forward transformations to determine gap *g* and row phase *r* from the input energy *E*_in_ and angle θ_in_, and *E* and Θ are the reverse transformations to obtain the energy and angle readback values. Three-dimensional magnetostatics computer codes (Chubar *et al.*, 1998 ▸) can be used to numerically calculate the magnetic field generated by a given gap and row phase configuration. Subsequently, the near-field synchrotron radiation emission from an electron passing through this generated magnetic field can be computed using an approach based on retarded potentials, and propagated by applying scalar diffraction theory *via* Fourier optics methods (Chubar & Elleaume, 1998 ▸). The polarization state of the emitted radiation is finally extracted from the propagated electric field. However, the inverse problem of determining the magnetic field, and hence the undulator parameters, to produce photons of a specified energy and polarization state is more challenging. A typical approach requires simulating a subset of the possible undulator configurations to predict the resulting photon energy and polarization state. This forms a look-up table which is used to select the appropriate gap and row phase for the input energy and angle. However, the simulated set of undulator parameters is sparse compared with the possible configurations and not evenly spaced in angle/energy, so interpolation of the results is necessary. An example of such a parameter set is shown in Fig. 1 ▸. The accuracy of the inverse transform from (gap, row phase) back to (energy, angle) is highly dependent on the density of the original grid of simulated undulator parameters, but simulation of all possible configurations is not feasible given the time-consuming nature of the computation. In the example given here, the linear arbitrary angles close to 0° (linear horizontal) and 90° (linear vertical) see large variation in the parameter values so calculation of the interpolated values in these regions is relatively less accurate. It is also unlikely that the state of the photon beam generated by a given set of undulator parameters will correspond precisely with that predicted by the theoretical calculation. Many factors related to the undulator can alter the polarization state which is observed at the endstation: inhomogeneity of the magnetic fields, small offsets in the magnet arrays, offsets in the electron beam trajectory, and off-axis alignment of the beamline acceptance. Other factors related to the beamline optics themselves may also contribute: steeper reflection angles (typically required for beamlines operating at energies below 100 eV), reflections in the energy range of the carbon edge due to carbon contamination of optics, manufacturing imperfections and misalignment may also impact the final polarization state.

Here, we present measurements of the photon beam generated by an APPLE-II undulator acquired using a high-precision soft X-ray polarimeter. A wide range of polarization states from different undulator configurations were systematically characterized to better understand how the observed polarization state deviates from that predicated by theory. Additionally, manual alteration of the gap and row phase parameters away from the table values was investigated to determine how the beam characteristics may be improved. Finally, the effect of altering the electron orbit through the undulator on the polarization state was also investigated. Such information is useful for the future development of the beamline and its capacity to deliver a high-quality photon beam with a precisely defined polarization state.

## 2. Experimental details   

The complete polarization measurements presented here were carried out on beamline I06 at Diamond Light Source, UK (Dhesi *et al.*, 2010 ▸). A schematic of the experimental setup is shown in Fig. 2 ▸. Photons are produced by an identical pair of APPLE-II type HU-64 undulators. Each undulator has two diagonally opposing magnet arrays which are movable in order to set the row phase, while the other pair remains in a fixed position; the position of upper and lower movable arrays are referred to as top/bottom row phase (TRP/BRP), respectively. Photon energy is controlled over the range 70–2100 eV by altering the gap between the magnet arrays, and altering the relative phase of the two magnet rows changes the polarization of the beam. All the following polarization states may be selected: linear horizontal (LH), linear vertical (LV), linear at arbitrary angle (LA), left-handed circular (LC), right-handed circular (RC) and elliptical, although LC/RC and LV are only available above 106 eV and 130 eV, respectively. The third undulator harmonic is utilized for energies above 1300 eV. The gap and row phase are set completely independently for each device so prompt polarization switching, for example from LC to RC, is possible by changing from one undulator to the other with differing settings (Bahrdt *et al.*, 2001 ▸; Quitmann *et al.*, 2001 ▸; Schmidt & Zimoch, 2007 ▸). Alternatively, they may be operated together, in conjunction with a phasing device (single-period undulator) located between them, which allows for greater photon flux. Each undulator is 2.11 m in length, has 33 periods of period length λ_u_ = 64 mm and can achieve a minimum gap between the magnet rows of 15 mm. During the experiment, only one undulator was in use during any given measurement (primarily downstream); the gap of the other undulator and phasing unit remained at their maximum values so that they had no influence on the electron beam.

I06 consists of two branches which utilize a collimated plane-grating-monochromator (PGM) optical scheme (Follath & Senf, 1997 ▸). The measurements presented here were carried out on the branch line. The photon beam is collimated vertically (dispersion plane) by a cylindrical mirror before it passes through the PGM which contains 150 lines mm^−1^ (used here), 400 lines mm^−1^ and 1200 lines mm^−1^ gratings for low-energy, high-energy and high-resolution measurements, respectively. A toroidal mirror downstream of the PGM focuses the beam to the exit slit. A second toroidal mirror is used for re-focusing to a user-provided endstation. All the beamline mirrors and plane gratings have gold coating. Here, the grazing angles of incidence are sufficiently small that the polarization effect upon reflection may be neglected: the ratio of s- and p-polarization, determined using the reflectivity calculation software *REFLEC* (Schäfers, 2008 ▸), is *R*_s_/*R*_p_ ≃ 1.006% for all the mirrors and *R*_s_/*R*_p_ ≃ 1.012% for the grating at 375 eV.

The high-precision Diamond polarimeter employed to carry out these measurements is a multilayer-based system containing a transmission phase retarder and reflection analyzer (Wang *et al.*, 2011 ▸). These are mounted in two azimuthally rotating stages which allow the retarder (α) and analyser (β) to rotate independently about the optical axis of the beam. Each azimuthal stage has an additional rotary stage which allows the multilayer incidence angles (retarder θ_R_, analyser θ_A_) to be tuned to the relevant Bragg angles. A photodiode is used to measure the light reflected from the analyser and is itself mounted on a rotating arm whose angle θ_D_ with respect to the optical axis is usually set to twice the analyser tilt angle. The mechanics all lie within a vacuum vessel that sits atop a hexapod providing six degrees of freedom for alignment. Prior to measurement with X-rays, the polarimeter is coarsely positioned and pre-aligned to the beam axis using externally mounted fiducial markers for reference with a laser tracker. Following this, fine alignment is achieved using X-rays. Low-level instrument control is performed *via* an Experimental Physics and Industrial Control System (EPICS) interface (Dalesio *et al.*, 1994 ▸). However, the user interface and high-level control is provided by the *Generic Data Acquisition* (*GDA*) software (Enderby & Pulford, 2004 ▸; Gibbons, 2008 ▸). This is the standard data acquisition interface at Diamond Light Source and allows for all the scanning to be performed *via* Python scripts.

A single polarization measurement is normally carried out by detecting the light intensity incident upon the photodiode at many α and β angles, for example every 10°, covering a full 360° rotation of each axis. To reduce the total acquisition time, only eight positions (every 45°) are currently measured for one of the rotations, usually α, as this is sufficient for fitting. However, the future introduction of fly scanning, where data are recorded during the motor movement, will allow a much greater number of angular positions to be sampled with no sacrifice in acquisition time. To minimize the impact of angular misalignment on the fitting results, the data for opposing angles of this rotation (0 and 180°, 90 and 270°, *etc*.) are averaged since they are equivalent. Furthermore, since each half of the complete 360° rotation are equivalent, the acquisition time can be further reduced by another factor of two by limiting the measurement range of β to 0–180°. Finally, the scanning direction of each motion is optimized so no time is wasted returning to the start point each time. Through these optimizations, a complete measurement of a single polarization can be completed in approximately 15 min. A standard automatic alignment procedure was used to provide consistent angular alignment below 50 µrad before measurements were carried out.

At each combination of α and β the normalized intensity is characteristic of the photon polarization state as described by the theoretical calculation (Wang *et al.*, 2012 ▸). By fitting this equation to the measured data, the so-called Stokes–Poincaré parameters *P*_1_, *P*_2_ and *P*_3_ can be extracted. These parameters can take values from −1 to 1 and describe the contribution of linear, linear at 45° and circular components to the overall polarization. Together, these parameters completely describe the polarization state of the light. Previously, this analysis was carried out using a tool developed in Igor Pro 6.32A (Hand *et al.*, 2016 ▸), but an improved analysis routine has now been developed in Python. This allows for data sets from multiple measurements to be fitted in a single batch script, dramatically speeding up analysis times. Since Python is also the scripting language used by *GDA*, it will be possible in the future to integrate this routine into the data acquisition scripts so that measured data can be automatically analysed and the polarization state immediately presented to the user.

Both polarizing elements used to carry out these measurements were Cr/Sc multilayers (number of periods *N* = 400, periodic thickness *d* = 2.57 nm, Cr/Sc thickness ratio Γ = 0.5) optimized for use at 375 eV. The optimized tilt angle for the phase retarder has previously been determined to be θ_R_ = 40.3° with a maximum phase shift Δ = −45.9° (Hand *et al.*, 2016 ▸). The s-component and p-component transmissions of the phase retarder at 375 eV are *T*_s_ = 0.25% and *T*_p_ = 0.8%, respectively. The analyser s-component reflectivity *R*_s_ is 26% and the p-component reflectivity *R*_p_ is 1.2%.

## 3. Results   

**Figure 3:**
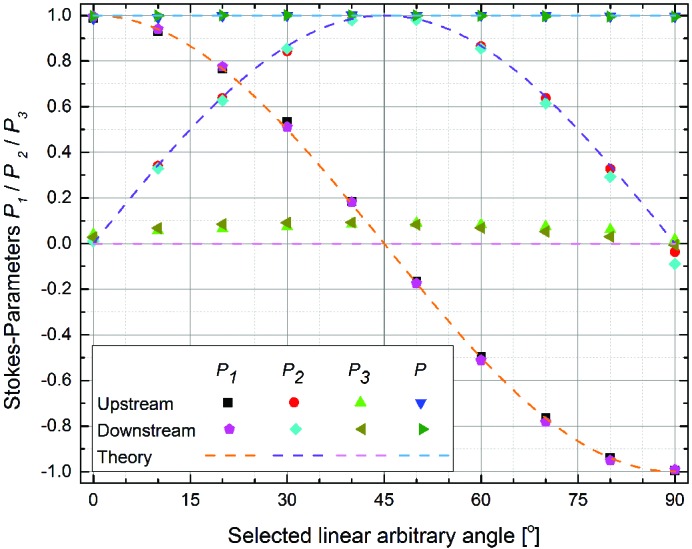
Stokes–Poincare parameters *P*_1_, *P*_2_ and *P*_3_ from polarization measurements of light emitted by the downstream and upstream undulators at 375 eV. The polarization fraction 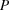 = 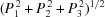 is also shown. Comparison with the predicted values determined from simple theory indicates that the expected linear light emitted by both undulators is slightly elliptically polarized.

A set of complete polarization measurements of a 375 eV photon beam emitted from the downstream undulator on I06 was carried out. Data were acquired when the undulator was set to emit linearly polarized photons at a range of arbitrary angles covering the entire range between LH (0°) and LV (90°). It is clear from the results in Fig. 3 ▸ that there are some discrepancies between the expected Stokes–Poincaré parameters which vary over the range of arbitrary angles. While the general shape of the curves for the linear components *P*_1_ and *P*_2_ is correct, a comparison with the theoretical values shows a clear, albeit small, deviation from the expected values for *P*_2_, especially for angles above 45°: the largest deviation is found at LA80° where the measured *P*_2_ differs from theory by ∼17%. However, more significant is the presence a non-zero *P*_3_ contribution, with a maximum value of *P*_3_ = 0.09 at arbitrary angles around 45°, which is not expected for purely linearly polarized light (*P*_3_ = 0 for all angles), *i.e.* the light is slightly elliptically polarized. For the downstream undulator these observations are consistent with previous measurements that have been carried out at 375 eV and also at 712 eV (Hand *et al.*, 2016 ▸). Identical measurements were also made using the upstream undulator; however, since the calibration tables for this undulator were not available, the magnet array row phases were manually set to values taken from the calibration table of the downstream undulator. The gap was then scanned to find the peak flux before proceeding with each measurement. Comparison of the results from the upstream undulator with those from the downstream undulator in Fig. 3 ▸ indicates that the same *P*_3_ contamination is present while the *P*_2_ values are closer to the expected values at angles above 45°: the maximum observed deviation of *P*_2_ in this case is <5%.

**Figure 4:**
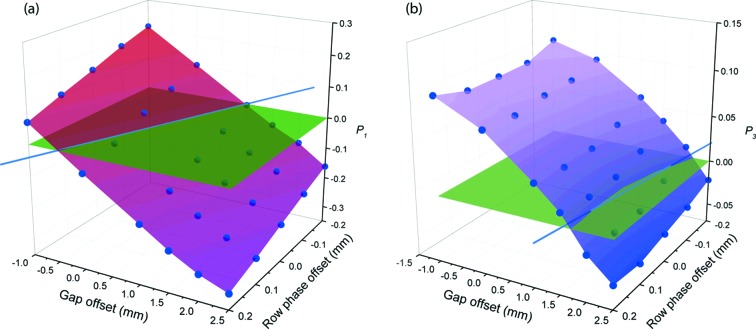
The (*a*) *P*_1_ and (*b*) *P*_3_ Stokes–Poincaré parameters plotted as a function of row phase and gap offset from their nominal values at LA45° form surfaces which intersect the plane (green) of *P*_1_ = *P*_3_ = 0 (the expected values at LA45°). The best-fit lines of intersection (blue) for the two parameters cross at a common value of the row phase and gap offset which provides an undulator setting that should minimize both *P*_1_ and *P*_3_ simultaneously.

One explanation for these deviations is some misalignment of the undulator magnet arrays which would affect the calibration of the positions (gap and row phase) required to achieve a given photon energy and polarization angle. However, given that the observed contamination is extremely similar in light generated by both the upstream and downstream undulators, it would seem unlikely that the same misalignment is present in both undulators. Nevertheless, complete polarization measurements were carried out to observe how deliberately altering the phase of a single magnet array and the gap of the downstream undulator affects the beam polarization. The undulator gap and row phase were initially set to their nominal values (TRP/BRP = ±18.1325 mm, gap = 22.4934 mm) for LA45° from the calibrations tables, as discussed in §1. The TRP was then moved to several positions covering a range of ±0.2 mm about the nominal values while the BRP remained fixed. At each TRP the undulator gap was also altered, covering a range of −1.0 mm to +2.5 mm from the nominal gap. The beam polarization was determined for each of these configurations as shown in Fig. 4 ▸.

As expected, the Stokes–Poincaré parameters were shifted from their nominal values for different undulator configurations and it was observed that the *P*_1_ and *P*_3_ parameters (both expected to be zero at LA45°) were particularly sensitive to the changes. When plotted against the row phase and gap values it was found that both parameters approximate a plane which intersects the plane representing *P*_1_ = *P*_3_ = 0. A best-fit line of intersection between the two surfaces was determined for each parameter and the crossing point of the two lines provides a unique combination of gap and row phase which should minimize both parameters. This crossing point is found to lie at offsets of ΔTRP = +2.5 mm and Δgap = −0.6 mm. It is unrealistic that the magnet arrays could be misaligned by this margin since the laser tracker technology which was used to position the arrays with respect to external survey fiducials allows for positioning to a precision of better than 100 µm, and likely close to 50 µm. Further evidence is provided by simple analysis using theory describing the influence of undulator magnet arrays on photon beam polarization (Young *et al.*, 2002 ▸). In this case we assume the circular contamination is the result of an effective phase shift of the magnetic field within the undulator. The horizontal (*B*_*x*_) and vertical (*B*_*y*_) components of the magnetic field along the central axis of an APPLE-II undulator are described by
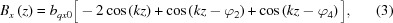


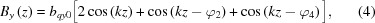
where 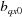 and 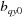 are the magnitudes of the horizontal and vertical components of the on-axis field generated by a single magnet array, *z* is the position along the axis of the undulator, 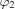 and 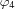 are the row phase shifts of diagonally opposing magnet arrays (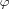 = 2πΔ*z*/λ) and *k* = 2π/λ with λ being the period of the magnet arrays. To generate linear light, 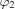 and 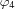 move by equal and opposite distances, *i.e.* 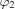 = 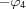 = 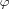, which leads to
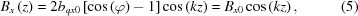


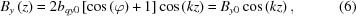
so the magnetic field components 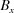 and 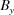 are in phase and the light is linearly polarized at an angle θ,
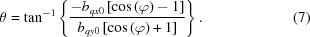
However, if there is an error in one (or both) of the row phase positions, *e.g.* 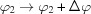, then the previous condition is broken, *i.e.* 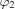 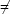 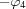. Consequently, *B*_*x*_ and *B*_*y*_ are no longer in phase and *P*_3_ becomes non-zero for any θ angles where *P*_2_ is non-zero, *i.e.* all angles other than linear horizontal and linear vertical (Koide *et al.*, 1991 ▸). This leads to a behaviour of the *P*_3_ component which matches that observed in the complete polarization measurements: the magnitude increases to a maximum as the angle is increased from θ = 0°, reaching a maximum at θ = 45°, before falling again and reaching zero at θ = 90°. The maximum value of *P*_3_ observed is *P*_3max_ = 0.075 which, in the theoretical description, corresponds to a row phase correction of ΔTRP = +0.8 mm. However, it has already been determined from the measurements above that ΔTRP = +2.5 mm would be required to minimize *P*_3_ for the I06 undulator. This indicates that, while it may be possible to minimize the *P*_3_ component by applying a (large) offset to the row phase position, the value of the offset differs considerably to that predicted by theory and points towards an additional influence on the polarization that increases the circular contamination of the linear light. A fundamental assumption of the above description is that the electron beam path is coincident with the central axis of the undulator magnet arrays. If in reality the electron beam is passing through the undulator slightly off-axis, this could have a significant impact on the photon beam polarization.

**Figure 5:**
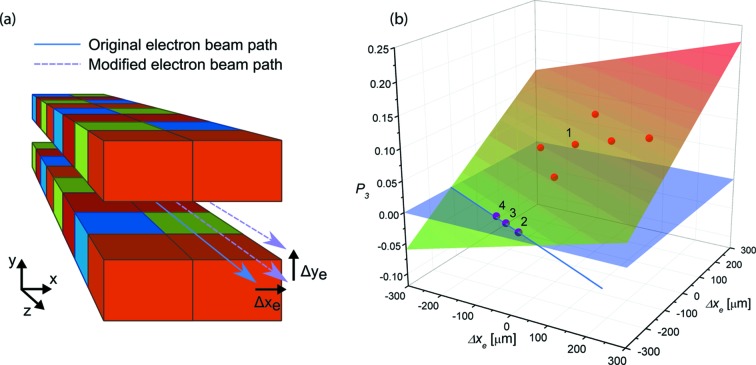
(*a*) Measurements of the photon beam polarization were carried out for different horizontal 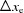 and vertical 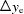 offsets of the electron beam orbit through the downstream undulator while the gap and row phase were set to produce LA45° light. (*b*) Similarly to the case where offsets in the top row phase and gap were introduced, when the *P*_3_ parameter is plotted against the offset coordinates 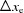 and 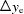 a plane is formed which intersects the plane representing *P*_3_ = 0 and provides a set of configurations which minimize *P*_3_.

A series of measurements were carried out in cooperation with the accelerator physics group to investigate how the orbit of the electron beam through the I06 undulators impacts the beam polarization characteristics. Polarization analysis was performed using the nominal undulator configuration for LA45° (as above) with different combinations of horizontal 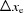 and vertical 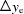 displacements of the electron beam orbit (see Fig. 5*a* ▸). Shifts of the electron orbit were achieved by altering the zero-point offsets of the beam position monitors before and after the undulator and allowing the corrector magnets, which work to maintain the electron beam orbit along a fixed path, to compensate for the change. Thus, the electron beam path is forced away from its nominal orbit in a stable and controllable manner.

It was discovered that the *P*_3_ parameter is particularly sensitive to the electron orbit offset and, similarly to the previous alteration of the row phase and gap offset, the results were found to form a plane which intersects the plane representing the expected value of *P*_3_ = 0 (see Fig. 5*b* ▸). In this case, the other Stokes–Poincaré parameters were insensitive to alteration of the electron beam path so no unique combination of horizontal and vertical beam offsets can be found to minimize *P*_3_. Instead, we find a set of solutions which lie along the intersection line. Following this, the electron beam was moved to several arbitrary coordinates described by this line and polarization measurements confirmed that they all minimize *P*_3_ as expected (*P*_3_ < 0.002 in all cases). A summary of these measurement results is shown in Table 1 ▸.

The minumum offset required in either the horizontal or vertical directions required to find a solution is found to be at least ∼160 µm; however, it is unlikely that there is an error in the beam orbit of this magnitude. A more likely scenario would be that the undulator itself is slightly mispositioned by of the order of 100 µm. While the undulator magnet arrays are positioned with respect to the external mechanics to probably better than 100 µm as previously described, and the orbit of the electron beam itself is known to an extremely high degree of precision in its own reference frame, the relative co-alignment of these two coordinate systems is less certain. As such, it is not unreasonable to expect that there could be such a misalignment between the electron beam path and the undulator axis on the scale described here.

## 4. Conclusion   

High-precision soft X-ray polarization analysis has been carried out using the Diamond polarimeter deployed at the I06 Nanoscience beamline at Diamond Light Source. Measurements of the linearly polarized beam at a range of arbitrary angles reveal a discrepancy between the observed and expected values for the Stokes–Poincaré parameters, specifically a non-zero *P*_3_ parameter which is expected to be zero for all linear polarizations. This behaviour, previously observed only in the downstream undulator (Hand *et al.*, 2016 ▸), is now also confirmed to exist when using the upstream undulator under identical conditions. The possibility of row phase position mis-calibration was investigated by deliberately offsetting one of the magnet arrays, but the required mis­alignment of ΔTRP = +2.5 mm to correct *P*_3_ is judged to be unreasonably large for this to be the sole cause of the issue. Finally, a set of polarization measurements were carried out to test the impact of altering the electron beam orbit through the undulators. Similarly to the row phase and gap offset measurements, a set of electron beam offsets were found to correct the *P*_3_ discrepancy, but the minimum required orbit offset of ∼160 µm is rather large. However, given that both the row phase and electron beam offsets investigated here clearly contribute to some degree, it is likely that complete elimination of the circular component from the linearly polarized photon beam would ultimately require modification to both aspects.

These measurements extend our understanding of the mechanisms which contribute towards contamination of linear­ly polarized light by an unwanted circular component and help us quantify their impact on the final polarization state. The ultimate goal of this work is to systematically identify and correct all the underlying sources of polarization contamination within the beamline system, but due to limitations of time and resources an alternative approach (Bahrdt *et al.*, 2010 ▸) may also be employed in the future to more quickly obtain the beam states required by users of the beamline. Nevertheless, it is already planned for additional simulations of the undulator sources to be carried out to better understand the contribution of an off-axis electron beam on the polarization contamination.

## Figures and Tables

**Table 1:** Comparison of Stokes–Poincaré parameters measured at electron beam offset positions Δ*x*_e_ and Δ*y*_e_ predicted to minimize *P*_3_ with those from the unshifted beam The column ‘Index’ refers to the corresponding numeric labels for the points in Fig. 5(*b*) ▸. Even in the worst case, the value of *P*_3_ is reduced by a factor of nearly 60 by shifting the electron beam.

	Electron orbit offset				
Index	Δ*x*_e_ (µm)	Δ*y*_e_ (µm)	*P*_1_	*P*_2_	*P*_3_
1	0	0	0.000	0.994	0.084
2	0	−260	0.000	1.001	0.000
3	−51	−235	−0.001	1.001	0.001
4	−92	−214	−0.003	1.007	0.001
